# Heart rate variability as a marker of multiple organ dysfunction syndromes: a systematic review

**DOI:** 10.1007/s10877-025-01296-w

**Published:** 2025-04-21

**Authors:** Anne Wojtanowski, Maxence Hureau, Mathieu Jeanne, Côme Bureau, Morgan Recher, Julien De Jonckheere

**Affiliations:** 1 CHU Lille, CIC IT 1403, 59000 Lille, France; 2 Univ. Lille, ULR 2694 METRICS, 59000 Lille, France; 3 Anesthesia and Intensive Care Department, CHU Lille, 59000 Lille, France; 4 Univ. Lille, ULR 7365 GRITA, 59000 Lille, France; 5 CHU Lille, Service de Médecine Intensive-Réanimation, 59000 Lille, France; 6 Pediatric Intensive Care Unit, CHU Lille, 59000 Lille, France

**Keywords:** Multiple organ dysfunction syndrome, Heart rate variability, Intensive care unit, Mortality, Autonomic nervous system

## Abstract

Multiple organ dysfunction syndrome (MODS) can be caused by many factors. Assessments of the severity of MODS are currently based on occasional measurements of several clinical variables (laboratory data, vital signs, etc.). The analysis of heart rate variability (HRV) as a guide to autonomic nervous system activity might be of value in the continuous assessment of the severity of MODS. We systematically reviewed publications on the value of HRV variables for the diagnosis of MODS in patients of any age admitted to the ICU. Two investigators independently searched the PubMed, Embase, Cochrane and Science Direct databases for articles in English or French published between 2004 and 2024. Ten studies were included and rated for endpoint bias (MODS or mortality), using the revised Quality Assessment of Diagnostic Accuracy Studies. Nine studies assessed MODS, and six assessed mortality. All the studies evidenced low HRV in patients with MODS and in non-survivors. The results of our review show that HRV indices are influenced by the severity of MODS and might serve as a tool for predicting mortality in patients with MODS. However, patient characteristics, and treatments and HRV processing methods must be taken into account when interpreting the results. In order to clarify the impact of MODS on HRV variables, methodologically rigorous studies are now needed.

## Introduction

In the intensive care unit (ICU), multiple organ dysfunction syndromes (MODSs, defined as the dysfunction of two or more organ systems) are associated with a high mortality rate because of the inflammatory immune response. MODS has various triggers; systemic infection, trauma, burns, cardiac arrest, and acute pancreatitis [1].

Various scoring systems are used to assess the severity of a disease and its consequences on various organ systems in patients admitted to the ICU. These scores facilitate monitoring of the patient’s condition during treatment. For example, the Sequential Organ Failure Assessment (SOFA) [2] score is supposed to be calculated daily and is generally calculated on inclusion in clinical studies as a guide to organ failure, which is directly correlated with the mortality rate. The Acute Physiology and Chronic Health Evaluation II (APACHE II) [3] score and the Multiple Organ Dysfunction Score (MODS) [4] can also be used to evaluate the severity of disease in adult patients in the ICU. In pediatric ICUs, the pediatric SOFA (pSOFA) score and the Paediatric Logistic Organ Dysfunction 2 (PELOD 2) score [5] are commonly used.

All the above-mentioned scores are based on vital signs (e.g. blood pressure, ratio of arterial partial pressure of oxygen and inspired oxygen fraction, etc.), laboratory variables (e.g. the platelet count, plasma levels of creatinine and bilirubin), and the use of life-support drugs (e.g. norepinephrine and adrenaline). Some scores also take account of the patient’s age or the reason for ICU admission. In clinical practice, the data required for score calculation are sometimes difficult to obtain, and so daily calculation of these scores for monitoring a patient’s clinical course is not practical. Moreover, a patient’s clinical course is sometimes marked by life-threatening complications that are missed by infrequent scoring. This is why novel, prospective tools for the continuous evaluation of organ dysfunction and the risk of death might help clinicians to rapidly and regularly evaluate a patient’s clinical course, his/her response to treatments, and the occurrence of complications during the stay in the ICU.

Heart rate variability (HRV) is a non-invasively assessed measure of autonomic nervous system activity and has been shown to reflect a patient’s wellbeing or stress under various conditions [6, 7]. Several HRV indexes have been described and evaluated in several clinical settings (Table 1) [8]. In patients with sepsis and septic shock, an early decrease in the magnitude of several HRV measurements is known to be related to the severity of the disease and the risk of organ failure. [9–11].

**Table 1:** HRV variables [8]

HRV indices	Summary
Time domain	
Mean	The mean R-R interval
SDNN	The standard deviation of the normal-to-normal (N–N) interval beats intervals represents the total signal variability. It evaluates autonomic nervous system global activity
SDANN	The standard deviation of the average N–N interval indicates very low signal variability
RMSSD	The root mean square of successive differences between adjacent N–N intervals corresponds to short-term variations that reflect parasympathetic nervous system activity
pNN50	The percentage of successive R-R intervals that differ by more than 50 ms is related to parasympathetic activity
Spectral domain	
LF	Low frequency (LF) is based on spectral analysis of the R-R series: the standard bandwidth in adults is 0.05–0.15 Hz. LF contains both sympathetic and parasympathetic modulations and is mainly influenced by baroreflex activity
HF	High frequency (HF) is based on spectral analysis of the R-R series: the standard bandwidth in adults is 0.15–0.4 Hz. HF contains parasympathetic modulation only and is mainly influenced by respiratory sinus arrhythmia
VLF	The very low frequency (VLF) variable is based on spectral analysis of the R-R series: the standard bandwidth in adults is 0.003–0.04 Hz. VLF reflects sympathetic, parasympathetic and humoral modulations
HFn.u. (also referred to as nHF)	The normalized HF content is defined as HFn.u. = HF/(LF + HF) and reflects parasympathetic activity
LFn.u. (also referred to as nLF)	The normalized LF content is defined as LFn.u. = LF/(LF + HF) and reflects variations in LF. The LF/HF ratio is often considered to be a marker of the sympathovagal balance
LF/HF	The LF/HF ratio is a marker of sympathovagal balance
Complexity domain	
ApEn	Based on probabilistic concept, approximate entropy is related to the regularity and complexity of a time series
SampEn	As a modification of approximate entropy, sample entropy is used to assess the complexity of physiological and other time-series signals
Non-linearity	
DFA	Detrended fluctuation analysis (DFA) is computed as the root-mean-square fluctuations F(n) of integrated and R-R signals on several timescales and is used to quantify the unpredictability of a time series. DFAα1 reflects short-term fluctuations, and DFAα2 reflects long-term fluctuations
Poincaré SD1 and SD2	SD1 and SD2 are computed from the Poincaré plot of the N–N interval against the prior interval. A Poincaré plot can be modeled as an ellipse, of which SD1 and SD2 are the diameters

The objective of the present study was to review the literature data on whether or not HRV analysis can be used to assess the severity of MODS in patients in the ICU.

## Materials and methods

The result of this systematic literature review were reported in accordance with the Preferred Reporting Items for Systematic Reviews and Meta-Analyses guidelines [12]. This review was registered on the International Prospective Register of Systematic Reviews (PROSPERO) under number CRD42024523557.

### Eligibility criteria

We sought to include original research articles published in English or in French between 2004 and 2024 and that covered patients of any age admitted to hospital and who then developed (or not) MODS. Studies of patients with chronic organ failure were excluded.

### Search strategy

The review addressed the following research question: “How might HRV analysis help to diagnosis MODS in patients in the ICU?” We searched the Cochrane, PubMed, Embase and Web of Science databases between March and April 2024, using the following query: (“multiple organ failure” OR “multiple organ dysfunction”) AND (“heart rate variability” OR “autonomic nervous system”) AND “intensive care”.

### Selection process

Two investigators (AW and JDJ) independently screened the publications with regard to the inclusion and exclusion criterion. Any disagreements were resolved by a third investigator (MJ).

### The data collection process and data items

Data on the study design, the country where the clinical trial was performed, the sample size, the patients’ condition, the HRV variables studied, the outcomes, the reference standard and the other study results were extracted by the screening team (AW and JDJ) and a third investigator (MH).

### Assessment of possible study bias

The risk of bias was independently assessed by two authors (AW and JDJ) on the QUADAS-2 scale [13]. Any disagreements were resolved by other investigators (MR or MJ).

### Effect measures

The values reported here are those given in the publications.

## Results

### Study selection

**Fig. 1:**
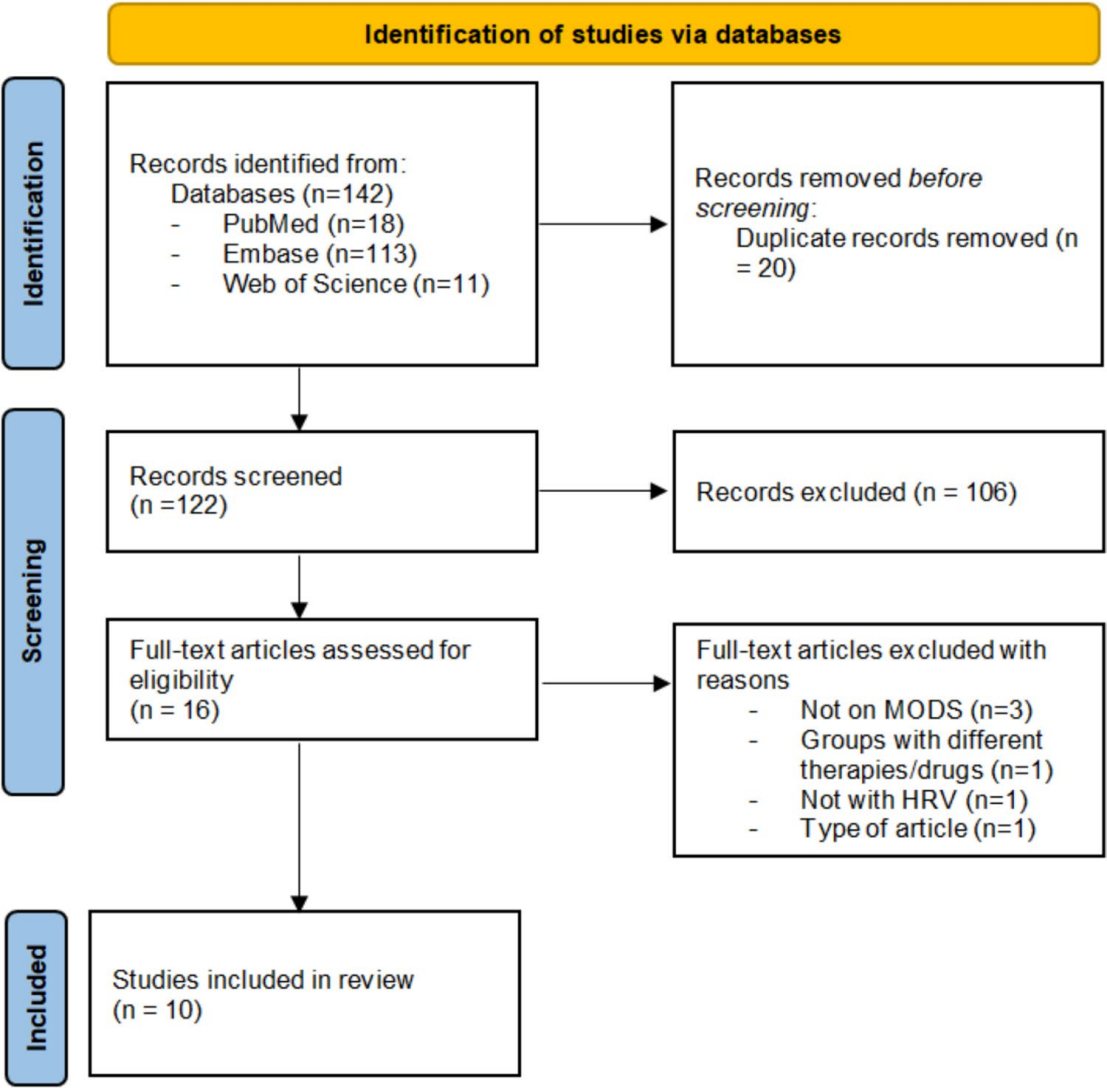
Flow diagram

The database search gave a total of 142 hits. After the removal of duplicates, 122 records were assessed for eligibility. Next, 16 full-text articles were then assessed for eligibility. Ten of the 16 were included in this review (Fig. 1).

### Characteristics of the studies

**Fig. 2:**
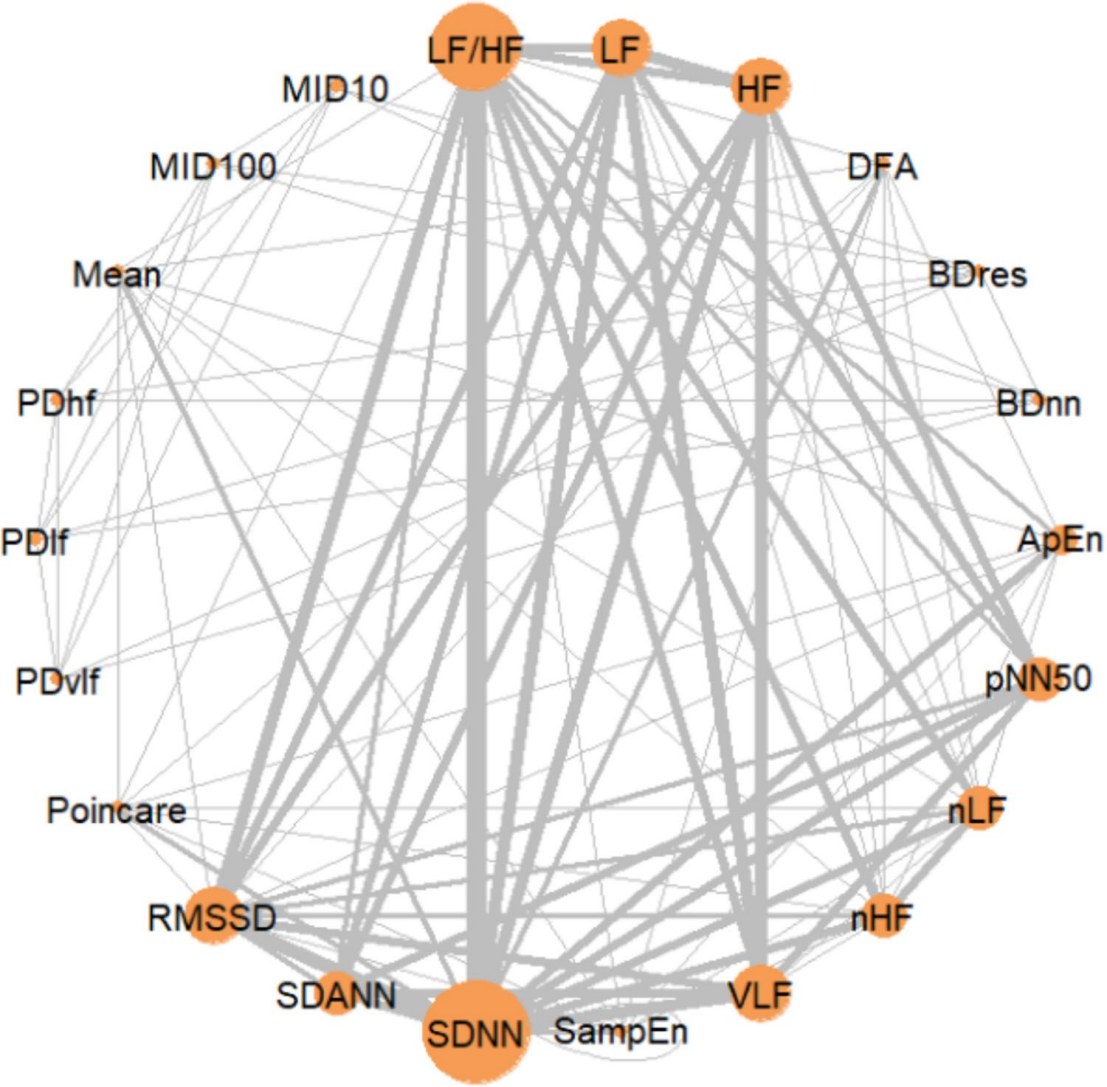
Network plot of the use of and comparisons of HRV indices. LF: low frequency; HF: high frequency; DFA: detrended fluctuation analysis; BD^RES^: AIF decay of a resampled R-R series in 5-min windows; BD^NN^: AIF decay over 1024 heart beat windows; ApEn: approximate entropy: AIF: automatic information flow; pNN50: the percentage of successive R-R intervals that differ by more than 50 ms; nLF: normalized low frequency; nHF: normalized high frequency; VLF: very low frequency; SampEn: sample entropy; SDNN: standard deviation of normal to normal; SDANN: standard deviation of the averages of normal to normal; RMSSD: root mean square of successive differences; PD^VLF^: AIF decay with regard to the predominant peak after R-R series filtering in the VLF domain; PD^LF^: AIF decay with regard to the predominant peak after R-R series filtering in the LF domain; PD^HF^: AIF decay with regard to the predominant peak after R-R series filtering in the HF domain; MID^100^: AIF decay over 100 s; MD^10^: AIF decay over 10 s

In 2005, Schmidt et al. studied autonomic dysfunction in 85 patients with MODS (mean ± SD APACHE II score: 37.8 ± 8) [14]. They measured various HRV indexes and compared them with normative data from the literature [8]. 61% of the patients were sedated, 62% received catecholamine, and 84% received ventilatory support. The 28-day overall mortality rate was 35%. The standard deviation (SD) of the normal-to-normal (N–N) interval (SDNN), the standard deviation of the average normal-to-normal intervals (SDANN), the percentage of successive R-R intervals that differ by more than 50 ms (pNN50), and the root mean square of successive differences (RMSSD) were computed for the 24-h period corresponding to the APACHE II score. VLF, LF, HF and LF/HF data were computed in a 5-min moving window and averaged over 24 h. All the HRV variables except RMSSD were significantly below normal in patients with MODS. Schmidt et al. also studied the influence of sedation, mechanical ventilation, catecholamine administration, and age on autonomic function. The HRV values were similar when comparing sedated and non-sedated patients and when comparing patients receiving catecholamine and those not. Age did not have any influence, whereas mechanical ventilation was associated with significantly lower pNN50, RMSSD, VLF, LF and HF values. Lastly, Schmidt et al. studied the ability of HRV measure to predict 28-day mortality and found that VLF was the best predictor of mortality over APACHE II and the sepsis score, with an area under the receiver operating characteristic curve (AUC_ROC_) of 0.68 for the whole MODS population, and 0.8 for patients with cardiogenic triggered MODS only (n = 31, 11 of whom died within 28 days). The researchers concluded that autonomic dysfunction assessed by HRV analysis may have prognostic value in patients with MODS and that neither age, sedation nor catecholamine administration affected HRV. In contrast, mechanical ventilation tended to decrease HRV and might have been a cofounding factor. In a secondary analysis of the same cohort, Schmidt et al. (2008) looked at whether autonomic dysfunction had a longer-term prognostic impact (i.e. on 180- and 365-day mortality) [18]. The overall mortality rates after 180 and 365 days were 65% and 71%, respectively. The AUC_ROC_ values for VLF after 180 and 365 days were 0.7 and 0.65, respectively. In a third retrospective study conducted in 2014, Schmidt et al. compared this MODS population with patients admitted to the ICU with chronic heart failure. The researchers found (i) that the HRV was lower in patients with MODS than in patients with chronic heart failure, (ii) significant inverse correlations between the APACHE II score on one hand and SDNN, LF and VLF on the other (*r* = -0.470, p < 0.0001, *r* = -0.3, p < 0.001 and *r* = -0.51, p < 0.0001, respectively), and (iii) significant inverse correlations between CRP on one hand and HF and VLF on the other (*r* = -0.27, p = 0.004; *r* = -0.42, p < 0.0001, respectively). The researchers confirmed the lack of influence of sedation and catecholamine administration (Fig. 2, Table 2).

**Table 2:** Characteristics of the studies

First author (year)	Country	Study design	Number of patients in total (number with MODS)	Type of patient	HRV variables	Outcomes	Reference standard	Results
Schmidt et al. (2005) [14]	Germany	Prospective, observational	90 (90)	Studied at the bedside in the supine position, ventilated (83%), and sedated (62%)	pNN50, RMSSD, VLF, LF, HF, LF/HF ratio, SDNN, SDANN	MODS	HRV normal range	↓SDNN ↓SDANN ↓pNN50 = rMSSD ↓LF ↓HF ↓VLF ↓LF/HF
						Mortality rate	28-day	↓lnVLF ↓lnSDNN
Hoyer et al. (2006–1) [15]	Germany	Prospective, observational	86 (43)	Studied at the bedside in the supine position, ventilated (80%) and sedated (80%)	Signal complexity Short-term indices: (BD^NN^, BD^RES^, PD^HF^, PD^LF^, PD^VLF^) Long term indices: (MID^10^, MID^100^)	MODS	HRV healthy volunteers	↑BD^NN^ ↑BD^RES^ = PD^HF^ ↑PD^LF^ = PD^VLF^ ↓MID^10^ ↓MID^100^
						Mortality rate	28-day	↑BD^NN^ ↑PD^mHF^ ↑PD^LF^
Hoyer et al. (2006-2) [16]	Germany	NR	232 (36)	NR	Signal complexity (BD^NN^, BD^Res^, PD^HF^, PD^LF^, PD^VLF^, DEC^long^)	Mortality rate	28-day	↑PD^HF^ ↑PD^LF^
Papaioannou et al. (2006) [17]	Greece	Prospective, observational	53 [19]	Supine position, sedated (propofol) and analgesia (fentanyl)	LF/HF, SDNN, ApEn, DFAα2	MODS	SOFA score > 7 (or > 5 for the surgery group)	↓SDNN ↓ApEn ↓LF/HF
						Mortality rate	NR	↓ApEn
Schmidt et al. (2008) [18]	Germany	Retrospective	90 (90)	Studied at the bedside in the supine position, ventilated (83%) and sedated (62%)	pNN50, RMSSD, VLF, LF, HF, LF/HF ratio, SDNN, SDANN	MODS	Normal HRV range	↓SDNN ↓SDANN ↓pNN50 = rMSSD ↓LF ↓HF ↓VLF ↓LF/HF
						Mortality rate	28-day 180-day 365-day	↓lnVLF
Green et al. (2013) [19]	Canada	Prospective, observational	33 [33]	NR	continuous individualized multi-organ variability measurement (CIMVA), standard deviation (SD), RMSSD, CV (interval-based), DFA-overall, DFAα1, DFAα2, Poincare SD1, Poincare SD2, skewness, kurtosis, LF/HF, LFnu, HFnu, ApEn, SampEn	MODS	Low MODS (SOFA score 0–2) Medium MODS (SOFA score 3–7) High MODS (SOFA score > 7)	↑Kurtosis ↓all other indices
Schmidt et al. (2014) [20]	Germany	Retrospective	130 (65)	MODS group: ventilated (89.2%) and sedated (72.3%)	pNN50, RMSSD, VLF, LF, HF, LF/HF ratio, SDNN, ASDNN, SDANN	MODS	Normal HRV range	↓SDNN ↓SDANN ↓pNN50 ↓LF ↓HF ↓VLF
Zhang et al. (2014) [21]	China	Prospective, observational	41 [9]	Studied at the bedside, with pressure support (22%), severe acute pancreatitis	SDNN, RMSSD, VLF, HF, nHF, LF, nLF, LF/HF	MODS	No MODS MODS: failure of at least 2 out of 3 organ systems (cardiovascular, respiratory, and renal) SOFA score > 6	= SDNN = RMSSD = TP = VLF ↓LF = HF ↓nLF ↑nHF ↓LF/HF
						Mortality rate	NR	= SDNN = RMSSD = TP = VLF = LF = HF ↓nLF ↑nHF ↓LF/HF
Badke et al. (2021) [22]	USA	Retrospective	5116 (5116)	Mechanical ventilation (30.3%) Catecholamines (4.9%) Dexmedetomidine (3%) Albuterol (25.6%)	HRV integer (HRVi), HRV dysfunction (HRVD) score	MODS	NPMODS (new or progressive MODS) No NPMODS	↑HRVD
						Mortality rate	28-day	↑HRVD
Luo et al. (2021) [23]	China	Prospective, observational	210 (109)	Studied in the supine position when the patient had been stabilized with fluids and/or drugs	nLF, nHF, nLF/nHF	MODS	SOFA > 5	↑nLF ↓nHF ↑nLF/nHF
						Mortality rate	30-day	↑nLF ↓nHF ↑nLF/nHF

*pNN50* the percentage of successive R-R intervals that differ by more than 50 ms, *RMSSD* root mean square of successive differences, *VLF* very low frequency, *LF* low frequency, *HF* high frequency, *SDNN* standard deviation of normal to normal, *SDANN* standard deviation of the averages of normal to normal

BD^NN^: AIF decay over 1024 heart beat windows; BD^RES^: AIF decay of a resampled R-R series in 5-min windows; PD^HF^: AIF decay with regard to the predominant peak after R-R series filtering in the HF domain; PD^LF^: AIF decay with regard to the predominant peak after R-R series filtering in the LF domain; PD^VLF^: AIF decay with regard to the predominant peak after R-R series filtering in the VLF domain; MD^10^: AIF decay over 10 s; MID^100^: AIF decay over 100 s; PD^mHF^: high frequency, vagal related peak decay; DEC^long^: AIF decay over 100 s; ApEn: approximate entropy; DFA: detrended fluctuation analysis; LFn.u. (also referred to as nLF): normalized low frequency; HFn.u. (also referred to as nHF): normalized high frequency; SampEn: sample entropy; ASDNN: mean of the standard deviations of all NN intervals for all 5-min segments in 24 h; TP: total power

The same group investigated an innovative signal processing concept that was designed to increase the prognostic value of HRV analysis (Hoyer et al. 2006-1) [15]. This consisted in studying the autonomic information flow (AIF) in the crude R-R series and the same series filtered in the VLF, LF and HF ranges. AIF decay reflects the loss of information over time, is independent of time series amplitudes, and is related to a signal’s predictability and regularity; the more complex the time series; the slower the AIF decay [24]. Hoyer et al. studied the prognostic value of several AIF decay indexes in patients with MODS: (i) BD^NN^: AIF decay over 1024 heart beat windows, (ii) BD^RES^: AIF decay of a resampled R-R series in a 5-min window, (iii) PD^HF^: AIF decay with regard to the predominant peak after R-R series filtering in the HF domain, (iv) PD^LF^: AIF decay with regard to the predominant peak after R-R series filtering in the LF domain, (v) PD^VLF^: AIF decay with regard to the predominant peak after R-R series filtering in the VLF domain, (vi) PD^mHF^: AIF decay with regard to the mean AIF after R-R series filtering in the HF domain, (vii) PD^mLF^: AIF decay with regard to the mean AIF after R-R series filtering in the LF domain, and (viii) PD^mVLF^: AIF decay with regard to the mean AIF after series filtering in the VLF domain.

The AIF decay indices were calculated in patients with MODS and healthy subjects over 24 h, over 6 h during the day, and over 6 h during the night. The researchers observed significant differences between patients with MODS and control patients in BD^NN^, BD^RES^, and PD^HF^ (AUC_ROC_ = 0.902, 0.819 and 0.804 respectively). They also demonstrated that BD^NN^, PD^mHF^ and PD^LF^ were predictive of 28-day mortality in patients with MODS (AUC_ROC_ = 0.688, 0.746 and 0.777, respectively). These results were confirmed in another study of AIF in patients with cardiovascular disease, patients in the ICU, and patients with schizophrenia. The non-survivors in the MODS population had elevated PD^mHF^ and PD^mLF^ [16] values.

Papaioannou et al. (2006) studied the variance of R-R intervals (which is similar to the SDNN), HF, LF, LF/HF, ApEn, and DFA α2 in 53 patients in the ICU (19 having undergone surgical treatment only, 19 with pre-existing medical conditions and having undergone surgery, and 15 with pre-existing medical conditions) [17]. A 10-min ECG was recorded every morning from admission to ICU discharge, and the HRV variables were computed over a 128-s artifact-free period. Mean and minimum values were computed for the whole ICU stay, and each HRV index was log-transformed before analysis to ensure that the data were distributed normally. The survival rate was 73.8%. 64.2% of patients had a mean SOFA score of less than 7, and 84.2% had a length of stay of less than 7 days. Patients with a mean SOFA score < 7 had higher mean variance, mean LF/HF, and mean ApEn values (p < 0.001). The mean and minimum ApEn were significantly lower in non-survivors than in survivors (p = 0.04 and 0.01, respectively). The minimum variance and mean LF/HF ratio were also significantly lower in non-survivors than in survivors (p = 0.04).

Green et al. (2013) evaluated the usefulness of HRV and respiratory rate variability for tracking daily organ dysfunction, the onset of shock, and the resolution of respiratory failure [19]. The study population included 33 ICU patients with respiratory and/or cardiac failure and continuous ECG recording from intubation to discharge. Green et al. used an in-house software package to perform a “continuous and individualized multi-organ variability analysis (CIMVA) of most of the HRV indices described in the literature (computed as the average of all 5-min CIMVA windows per 24 h). Three groups of patients with MODS were defined: low MODS (a SOFA score from 0 to 2, 51 measurements), medium MODS (a SOFA score from 2 to 7, 106 measurements) and high MODS (a SOFA score > 7, 32 measurements). Both HRV and respiratory rate variability decreased with the severity of MODS. Although several HRV measures were able to detect the onset of shock, only respiratory rate variability was of value for detecting the resolution of respiratory failure. The two HRV variables that best discriminated between the three MODS groups were the coefficient of variation (CV = SDNN/mean N–N) and DFAα2 (p < 0.001). The SDNN and wavelength area under the curve varied significantly around the time of onset of shock.

Luo et al. (2021) performed a short-term spectral HRV analysis of trauma patients in the ICU [23]. The outcomes were 30-day mortality, the incidence of MODS (defined as a SOFA score > 5), and the length of stay in the ICU. Using a 5-min moving window, the researchers studied the normalized HF, LF and LF/HF data over the first 24 h of the patients’ stay in the ICU. 210 patients were included, and 40 patients died within 30 days. In the patients who died, nHF was significantly lower, whereas nLF and the LF/HF ratio were significantly higher. The LF/HF ratio was predictive of mortality, with an AUC_ROC_ of 0.924. In patients with MODS, nLF and LF/HF were significantly lower, whereas nHF was significantly higher. LF/HF was able to predict MODS, with an AUC_ROC_ of 0.826. Moreover, LF/HF and nLF were correlated with the length of stay in the ICU (r = 0.451 and 0.453 respectively).

Badke et al. (2021) looked at whether the Heart Rate Variability Dysfunction (HRVD) score (a novel, age-normalized index of autonomic nervous system dysregulation) was associated with the onset or progression of MODS or with death in critically ill children [22]. Continuous heart rate data were extracted from bedside monitors, using the BedMaster system (Excel Medical, Jupiter, FL). The HRV integer (HRVi) was computed as the standard deviation of the heart rate sampled every 1 s over 5 consecutive minutes. Patients included between January 2012 and August 2016 were included in the discovery cohort used to derive HRV cut-off values, whereas patients included after August 2016 were included in the validation cohort. The HRV data from the final 12 h of the stay in the pediatric ICU (PICU) by survivors in the discovery cohort was taken as the reference for calculation of a median normalized HRVi on day 1. To produce the HRVD score, this HRVi index was adjusted for age by subtraction of the median for the reference age group from the HRVi measurements during the first 24 h of the stay in the PICU and then division by the interquartile range. Two primary outcomes were assessed: the new or progressive MODS (NPMODS) and death. NPMODS was defined as (i) an increase in the pSOFA score of 4 or more points from day 1 to any time between days 2 and 7 of the stay in the PICU, (ii) an increase in the pSOFA subscore by 2 or more points from day 1 to any time between days 2 and 7 of the stay in the PICU (only in patients with a subscore of 0–2 on day 1), or (iii) a persistent subscore of 3 or more at some time between days 2 and 7 of the stay in the PICU (only in patients with an initial subscore of 3 or more on day 1). The 28-day in-hospital mortality rate was studied. 5116 and 2107 patients were included in the discovery and validation cohorts, respectively. The HRVD index on day 1 was able to discriminate between patients with NPMODS at some point in the day 2 to day 7 period and patients with no NPMODS (AUC_ROC_ = 0.67) and between survivors and non-survivors (AUC_ROC_ = 0.84).

Zhang et al. (2014) investigated HRV as a marker of infected pancreatic necrosis (IPN) or MODS in patients with severe acute pancreatitis [21]. They included 41 patients within 72 h of symptom onset and recorded a 5-min ECG for HRV analysis (VLF, LF, nLF, HF, nHF, LF/HF, SDNN, and RMSSD). Failure in any of three organ systems (cardiovascular, respiratory and renal) was documented when the organ’s SOFA score was 2. MODS was defined as the failure of two or three organ systems. Sixteen patients developed IPN, nine patients developed MODS, and four patients died. A low LF and LF/HF and a high nHF were observed in patients developing IPN or MODS. nLF was lower in patients with MODS but not in patients with IPN. The AUC_ROC_ for the prediction of MODS were 0.837 with nHF and 0.906 with LF/HF; these values were similar to the AUC_ROC_ for the APACHE II score (0.899).

When considering the 10 studies reviewed here, SDNN was the most commonly examined HRV index (n = 7), followed by LF/HF (n = 6), RMSSD (n = 4), LF (n = 4), HF (n = 4), VLF (n = 4), SDANN (n = 3), pNN50 (n = 3), nLF (n = 3), nHF (n = 3), and ApEn (n = 2) (Fig. 2). All the other HRV indexes were assessed in one article each.

### Risk of study bias

With regards to the risk of bias in evaluations of MODS (as rated on the revised Quality Assessment of Diagnostic Accuracy Studies (QUADAS-2) scale) [13], most of the reviewed studies included patients who had several clinical conditions, were sedated or were ventilated (Table 3). Hence, the risk of bias for patient selection was quoted as “unclear”. Papaionnou et al.’s study included only sedated, mechanically ventilated patients and so was rated as having a low risk of bias for patient selection. Most studies declared having followed the guidelines on the calculation of HRV variables issued by the Task Force of the European Society of Cardiology and the North American Society of Pacing and Electrophysiology (ESC-NASPE) and therefore presented a low risk of bias in this respect [8]. The few studies that apparently did not follow these guidelines were rated as having an “unclear” risk of bias. Whereas most of the studies used the SOFA score to discriminate between severe MODS and moderate/no MODS, Schmidt et al. and Hoyer et al. compared HRV data from a population of MODS patients with HRV data from a healthy population; this constituted a high risk of bias for the reference standard.

**Table 3:**
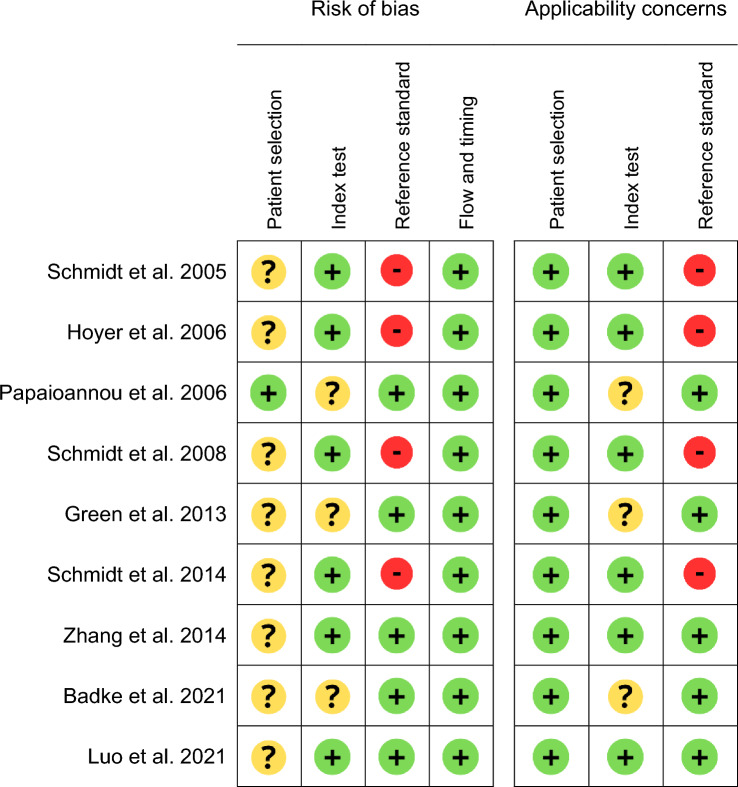
QUADAS2 study ratings for MODS

For the evaluation of mortality, the QUADAS-2 risks of bias of patient selection and the index calculation were the same as for MODS (Table 4). With regard to the reference standard, most of studies compared HRV with 28-day mortality. Papaioannou et al. and Zhang et al. did not define mortality and were therefore considered to have an “unclear” risk of bias.

**Table 4:**
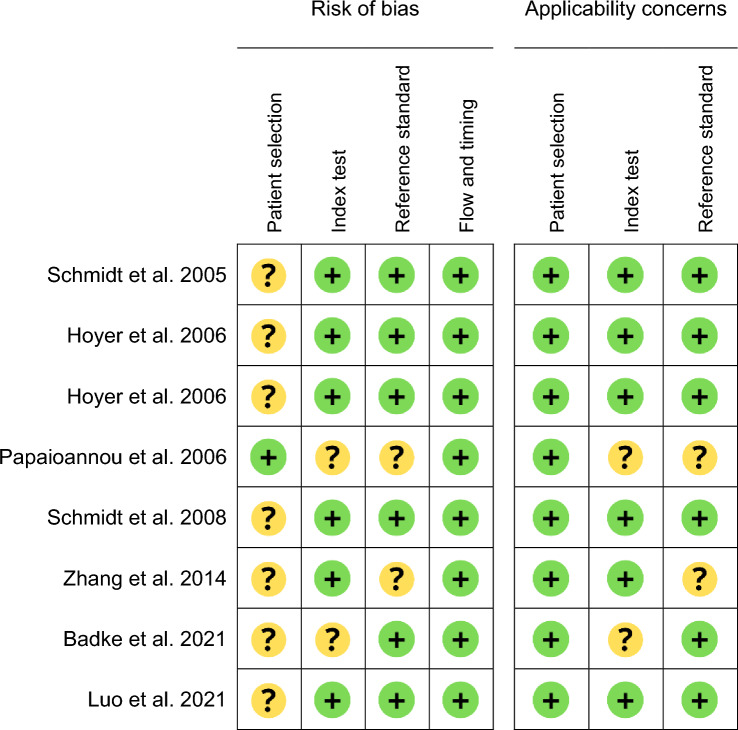
QUADAS2 study ratings for mortality

## Discussion

### HRV analysis

According to the 1996 ESC-NASPE guidelines, HRV analysis should be performed on a beat-to-beat basis on N–N intervals from an ECG signal [8]. For spectral analysis, the optimal ECG sampling rate range is 250–500 Hz; a lower sampling rate (100 Hz) may only behave satisfactory if an interpolation algorithm is used to refine the R peak. The duration of the HRV analysis window is also important. In many studies, SDNN and other time-domain analyses are computed over a 24-h window and are thus related to both short-term and long-term variabilities. However, depending on the study objectives, a narrower window (e.g. 5 min) may be appropriate. Spectral HRV analysis requires stationarity and should not be conducted in broad time windows. In practice, 2- to 5-min windows are acceptable. In order to standardize investigations of short-term HRV in a stationary system, 5-min recordings are preferable.

While most of the studies reviewed here claimed to have followed the ESC-NASPE guidelines strictly, several other studies appeared not to have done so. For example, Badke et al. did not compute their “HRV integer” index on a beat-to-beat basis but used the standard deviation of the heart rate sampled every 1 s over 5 consecutive minutes. Green et al. used a 125 Hz ECG sampling frequency but did not mention the use of an interpolation algorithm. Papaioannou et al. performed a fast Fourier transform spectral analysis in a 128-s moving window. These differences in the algorithms might have influenced the studies’ results and interpretations. Considering the studies as a whole, however, the results were relatively consistent.

Although VLF, LF and HF spectral components are often expressed as absolute values, they can also be quoted in normalized units (n.u.) HFn.u. and LFn.u. are the values of each spectral component, relative to the total power minus VLF: HFn.u. = HF/(HF + LF), LFn.u. = LF/(HF + LF), and HFn.u. + LFn.u. = 1. Hence, HFn.u. and LFn.u. represent the imbalance between the two branches of the autonomic nervous system. In Luo et al.’s study, the sum of the mean values of LFn.u. and HFn.u. was reported as 1 in each patient group (survivors, non-survivors, patients with MODS, and patients without MODS). In Zhang et al.’s study, however, the sum of the median LFn.u. and HFn.u. values was never 1; this raises questions about how the spectral components were normalized.

### HRV and MODS

Overall HRV variability (SDNN), spectral power, and signal complexity appear to decrease as MODS becomes more severe. The LF/HF ratio appears to best discriminate between patients with severe MODS and those with moderate MODS or no MODS. Badke et al. studied the value of HRV for diagnosing changes in the severity of MODS in the PICU; the HRV decreased in patients developing new or progressive MODS. In 2003, Pontet et al. made similar observation in ICU patients with sepsis [25]. A 10-min HRV analysis during the first 24 h in the ICU showed that patients who subsequently developed MODS had lower SDNN, RMSSD, LF power and HF power values. Other researchers studied HRV as a guide to changes in the clinical course in the ICU. In 1993, Garrard et al. studied changes in various HRV variables during and after septic syndrome in 17 ICU patients. The researchers demonstrated that LF power and the LF/HF ratio were lower during the septic phase [26]. Similarly, several studies conducted in neonatal intensive care units evidenced a loss of HRV complexity at the onset of sepsis [27, 28].

### HRV and mortality

Badke et al. reported that non-survivors had a low standard-deviation based HRVD index (AUC_ROC_ = 0.84). In Schmidt et al.’s study, both SDNN and VLF were predictive of 28-day mortality. Although VLF in short (≤ 5 min) recordings is of dubious value and should be avoided for the interpretation of HRV [8], the low overall variability (as expressed by the SDNN) in non-survivors with MODS is consistent with the other literature data. In 2006, Norris et al. demonstrated that SDANN was predictive of death in a cohort of 2088 trauma patients in the ICU (AUC_ROC_ > 0.7) [29]. In a systematic review in 2018, Castilho et al. concluded that low SDNN, RMSSD, LF, HF and LF/HF values were associated with mortality in patients with sepsis [11]. HRV signal complexity was also low in non-survivors and appears to be predictive of death in patients with MODS.

Despite concordant results for most HRV indices, our review highlighted contradictions regarding the LF/HF ratio. LF/HF was significantly lower in non-survivors (p = 0.04) in Papaioannou et al.’s study but was significantly higher in non-survivors in Luo et al.’s study. This difference might be due (at least in part) to inter-study differences in the inclusion criteria and the computed HRV variables. For example, the width of the moving window for spectral analysis was 5 min in Luo et al.’s study and 128 min in Papaioannou et al.’s study. Luo et al.’s use of a very narrow window for computation of a fast Fourier transform might have led to underestimation of the spectral content in general and the content in the LF domain in particular [8]. Papaioannou et al.’s study included patients with trauma, patients with pre-existing medical conditions, and patients recovering from surgery, whereas Luo et al.’s study included only patients with multiple trauma in an ICU. In Papaioannou et al.’s study, patients were ventilated, sedated (with propofol) and given analgesics (fentanyl), whereas Luo et al. did not specify their patients’ ventilatory and sedative status. There are conflicting opinions with regard to the effect of sedation on HRV. Schmidt et al. (2005) demonstrated that mechanical ventilation was associated with several significantly elevated HRV indices [14]. In contrast, Schmidt et al., did not highlight any influence of sedation and catecholamine administration on HRV. Bradley et al. studied the effect of discontinuing sedation on HRV in critically ill patients [30]. The researchers observed a significant increase of HRV after sedation interruption and found that the HRV increase was greatest in patients with mild and moderate MODS. Studies performed during surgery under general anesthesia demonstrated that hypnotic drugs affect HRV—notably through their sympatholytic effect [31, 32]. Huhle et al. demonstrated that propofol induction significantly reduced HRV features [33]. A patient’s sedative and ventilatory status may therefore influence HRV and should be taken into account when interpreting the results. However, most of the studies reviewed here included ventilated and non-ventilated patients and sedated and non-sedated patients; this might constitute a source of bias in interpretation of the results. Indeed, patients with severe MODS are usually sedated more deeply, which might be associated with low HRV. Among the studies reviewed here, only Badke et al. included the use of mechanical ventilation, sedation and catecholamine in a logistic regression analysis to determine the independent ability of HRV to predict the patients’ MODS status. In their review, Castilho et al. (2008) concluded that the heterogeneity of the study populations could be a bias and might give conflicting results for some HRV indices [11].

## Conclusion

Our review shows that HRV indices are influenced by the severity of MODS and might be useful tools for predicting the risk of death in patients with MODS. However, it is clear that the patients’ characteristics and the signal processing methods used for HRV computation must be taken into account when interpreting the results. Even though that HRV indices appear to be valuable tools for the diagnosis of ICU patients with MODS, a better understanding of how MODS affects HRV variables will require methodologically rigorous studies with regard to patient selection and signal processing.

## Data Availability

No datasets were generated or analysed during the current study.
